# Clinical, radiographic and biochemical assessment of proximal cavities restored with composite resin using incremental vs. bulk packing techniques: One-year randomized clinical trial

**DOI:** 10.1186/s12903-024-04746-0

**Published:** 2024-09-30

**Authors:** Monaliza Maher Abdelaziz, Shereen Fathy, Amany Ahmed Alaraby, Wessam Ibrahim Shehab, Marwa Mohamed Temirek

**Affiliations:** 1 Department of Conservative Dentistry, Faculty of Dentistry, October 6 University, 6th of October, 12563 Egypt; 2 Department of Oral Radiology, Faculty of Dentistry, October 6 University, 6th of October, 12563 Egypt; 3 Department of Oral Medicine, Periodontology & Oral Radiology, Faculty of Dentistry, October 6 University, 6th of October, 12563 Egypt; 4 Department of Conservative Dentistry, Faculty of Dentistry, Fayoum University, Fayoum, 63514 Egypt; 5 Department of Conservative Dentistry, Faculty of Dentistry, October 6 University, 6th of October, 12563 Egypt

**Keywords:** Bulk fill composite, Recurrent caries, Periodontal assessment, Radiographic assessment, MMP-9

## Abstract

**Background:**

Bulk-fill resin composites may suffer from recurrent caries around compound proximal restorations in posterior teeth, especially at the proximo-gingival interface.Over 12 months, will the bulk fill technique affect the caries recurrence rate at gingival margins when compared to the conventional incremental packing technique? How early will the first clinical, radiographical, and biochemical evidence of caries recurrence occur?

**Methods:**

After randomization, in 30 patients with two compound (OM or OD) supragingival lesions, one tooth was restored using the bulk fill technique on one side (group 1) (*n* = 15). In contrast, the other tooth on the other side was restored utilizing the incremental layering technique (group 2) (*n* = 15). Both teeth received restorative material (X-tra fil, Voco, Cuxhaven, Germany). The FDI criteria were used to evaluate restorations. As for the periodontal assessment, the gingival index, plaque index, papillary bleeding scoring index and periodontal pocket depth were evaluated. The gingival crevicular fluid (GCF) specimens were gathered, and MMP-9 was extracted and quantitated by ELISA. A customized radiographic template was designed, and 3D printed digital bitewing radiographs were taken. Assessments were done clinically, radiographically and biochemically at baseline (1 week) and after 3, 6 and 12 months. Data was statistically analyzed.

**Results:**

The null hypothesis was accepted clinically; no statistically significant differences appeared between bulk and incrementally filled posterior restorations. As for the radiographic assessment, the null hypothesis was accepted except for increased periodontal ligament width at 3 months. The null hypothesis for the biochemical evaluation was rejected as there were significant changes in levels of MMP-9 at different testing times.

**Conclusions:**

1. With similar results but less sensitivity and significant time saving, the bulk fill technique can be considered an efficient alternative to the incremental fill technique in restoring proximal cavities. 2. Early evidence of caries recurrence can be correlated to an increase in the MMP-9 level in gingival crevicular fluid, followed by an increase in radiographic periodontal ligament width measurement.

**Trial registration:**

An ethical approval from the Research Ethics Committee at the Faculty of Dentistry, October 6 University, (Approval No. RECO6U/5-2022). The study was registered at the Pan African Clinical Trials Registry on 24/07/2023 with an identification number (PACTR202307573531455).

## Introduction

Dental restorations play a substantial role in preserving periodontal health. Restoration type, margin adaptation, restoration contouring, and smoothness of surfaces all have vital biological influences on periodontal tissue [1]. Conservation of a healthful periodontium is mandatory for long-life successful dental restorations [2]. Proximal restorations may impact the health of the periodontium if the critical spaces between suprarenal connective tissue attachment and the junctional epithelium are not considered. Bone resorption and connective tissue attachment loss may occur due to deficient distance to preserve interproximal tissue health [2], so gingival healing is compulsory before dental restorations [3].

While resin composite is a popular choice for dental restorations, it is essential to be aware of its drawbacks [4]. The incremental layering of composite resin, with increments of ≤ 2 mm, has been proposed to minimize shrinkage stress, enhance the degree of conversion, and prevent breakdown at restoration margins [5]. However, it is crucial to note that this technique can lead to void entrapment between composite increments, bonding failure at the interface, and extended clinical chairside time [6]. Awareness of these potential issues is key to successfully using resin composites.

Therefore, bulk-fill resin composites arose with low stress of polymerization shrinkage, low and high viscosities, and increased polymerization depth, with the ability to restore the cavity in a single increment from 4 to 5 mm. The use of such a technique presents a shorter and simpler restorative procedure [7].

Recurrent caries around the margins of resin composites are one of the adverse effects of microleakage. Recurrent caries correlate with marginal and interfacial gap sizes and mechanical stresses during mastication [8].

According to the recommendations of the American Dental Association (ADA), posterior restorations perspective, a successful restoration can be determined by the absence of radiographic signs, according to the degree of caries risk of each patient [9, 10], indicating underlying carious lesions, voids, open margins, or overhanging [11, 12]. However, identifying the health condition of restoration, particularly for compound restorations, remains a significant challenge, especially at the proximo-gingival interface.

Several studies have revealed a significant issue in dental materials science: the stability of the hybrid layer delivered by bonding systems in watery environments. This instability is primarily due to the hydrolytic degradation phenomena of resins and collagen fibrils, which dissipate over time.[13] The endogenous MMPs attached to the organic dentin matrix can disintegrate the exposed collagen fibrils inside the hybrid layer if adhesive monomers do not cover them.[14]

Pathogenic microorganisms can cause periodontal damage and restoration degradation using a broad array of macromolecules, enclosing many proteins secreted by several cells, such as matrix metalloproteinases (MMPs), which are affected by tissue metalloproteinase inhibitors, or TIMPs, and are involved in the breakdown of the extracellular matrix [15]. Degeneration of extracellular matrix proteins (ECMs) by proteinases is a crucial aspect of periodontal disease and originated from bacteria in dental plaque or cellular sources [16]. Former investigations showed that proteases can activate MMPs, leading to periodontal breakdown [17]. Several studies showed elevated levels, in Gingival Crevicular Fluid (GCF), of MMP-9 and MMP-8 in patients with periodontal disease, and these molecules have been suggested to be valid indicators of the disease [18] and have been observed for 2 months [19, 20]. These proteolytic enzymes show increased levels in carious dentin lesions [21]. A significant association between MMP-9 in deep and shallow carious lesions was reported [22].

Over 12 months, in patients with compound proximal composite resin restorations in posterior teeth, we will explore a crucial question: does using the bulk fill technique significantly impact the caries recurrence rate at gingival margins when compared to the conventional incremental packing technique? This could revolutionize our approach to restorative dentistry.

Therefore, the current study’s null hypothesis supposes no significant clinical in assessing recurrent caries, periodontal, radiographical or biochemical difference after applying posterior restorations using two different packing techniques.

## Materials & methods

### Selection of subjects & study design

A randomized, controlled clinical trial, double-blind, split-mouth study design was performed. The patients who met the trial’s eligibility requirements were chosen among the frequent visitors to the clinics of Conservative Dentistry and Oral Medicine and Periodontology Departments, Faculty of Dentistry, October 6 University. Ethical approval was obtained from the Research Ethics Committee at the Faculty of Dentistry, October 6 University, (Approval No. RECO6U/5-2022). The study was registered at the Pan African Clinical Trials Registry on 24/07/2023 with an identification number (PACTR202307573531455).

### Sample size

A power analysis was planned to have sufficient power to apply a statistical test of the null hypothesis that there would be no difference between the tested groups regarding recurrent caries. The sample size was calculated based on statistical analysis using http://biomath.info/power depending on the previous study [23]. A sample size of sixty restorations (thirty restorations in each of the two groups). The total number of restorations was divided randomly into two groups.

### Inclusion and exclusion criteria

Patients with compound class II (OM or OD) supragingival lesions were selected for the current study. Examinations of patients included a medical and dental history and extra-oral and intra-oral examinations. The chosen patients (*n* = 30) were 18 to 50 years old, with fully erupted occluding permanent dentition having 2 carious lesions with proper contact with adjacent teeth, scoring based on the International Caries Detection and Assessment System (ICDAS) as code 4 or 5. Patients with complex class II, crowding, spacing, severe pain, tooth mobility, fistula, and any signs of severe periodontal disease or irreversible pulpitis were excluded [24]. Inclusion criteria also included patients with moderate caries index according to ADA Caries Risk Assessment, group function occlusion, healthy gingiva and normal sulcus depth.

**Fig. 1:**
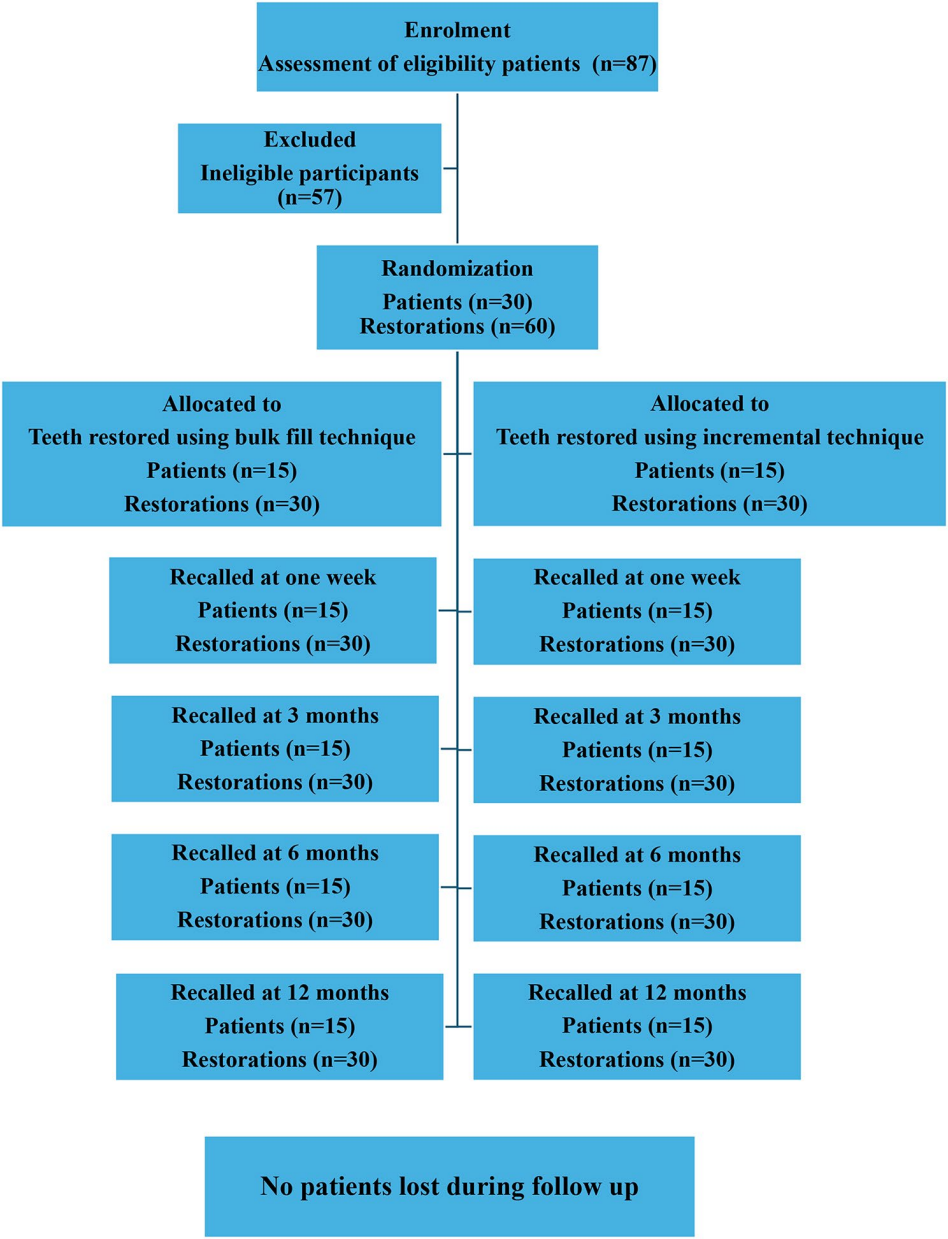
CONSORT flow diagram

Patients who met the eligibility criteria were registered in the study after giving their written informed consent, and they could leave the trial at any time during evaluation for any reason. A consort flow chart presents the flow of participants through each stage of the study (Fig. 1).

### Randomization method

The study involved 30 patients. Each patient received restorations on two opposing sides. One tooth was restored using the bulk fill method (group 1), while the other tooth on the other side received the incremental layering technique (group 2). Details of the randomization process, which ensured allocation concealment, are presented in Fig. 1. Block randomization with a random number table was used with a 1:1 allocation ratio. Odd numbers resulted in the first tooth being restored with a bulk filling, while even numbers resulted in the first tooth receiving an incremental layering technique. Participants and the examiner remained blind throughout the study to minimize bias [24].

The study’s reporting adhered to the Consolidated Standards of Reporting Trials (CONSORT) statement.

### Clinical procedure

Local anesthesia was administered, and a rubber dam was applied for isolation of the quadrant containing the carious tooth. Class II cavities were done using a high-speed handpiece (Dentsply Sirona, NA, USA) with a diamond fissure bur. All cavity preparations followed a conservative design, restricted to carious tissue, and undermined tooth structure removal. After the preparation, a sectional matrix system was applied (TOR VM dental manufacturing company, Moscow, Russia). A universal adhesive system (Futurabond U, Voco, Cuxhaven, Germany) was used according to manufacturer instructions, and then light curing was done for 10 s utilizing a woodpecker light cure unit (1200 mW/cm^2^).

X-tra fil (Voco, Cuxhaven, Germany) bulk-fill composite resin was used for both restoration techniques. For cavities restored utilizing the incremental layering technique, oblique increments were inserted. The increments were all kept within a 2-millimeter maximum thickness. The first increment was light-cured for 10 s at zero distance, followed by the insertion of another increment. For teeth restored using the bulk fill technique, an increment of 4 mm is placed in one layer inside the cavity using a Teflon-coated condenser, then light cured. The restorations were finished and polished using a two-step 3 M™ Sof-Lex™ F/P spiral wheel system and Ultradent Jiffy Hi Shine polisher cup. The proximal contact tightness was checked with waxed dental floss [25].

The evaluation of restorations was performed using the FDI criteria with different scores:

1. No secondary caries: clinically very good;
2. Very small and localized demineralization: clinically very good;
3. Large areas of demineralization: clinically sufficient/satisfactory;
4. Caries with cavitation: clinically unsatisfactory; and.
5. Deep secondary caries: clinically poor [26].

One blinded assessor (M.M.A.) evaluated the restoration. Restorations were examined visually under the overhead light of the dental unit after isolating the field using cotton rolls. All tooth surfaces were dried [44]. All restorations were further examined using a double-ended dental explorer (23/17A, Superior Instruments, United Kingdom) according to FDI criteria. Marginal discrepancies were checked using waxed dental floss for any roughness or irregularities.

### Determination of MMP-9 level in GCF

After isolation of the tooth with a cotton roll, the plaque was removed supragingival with a curette without contacting the gum margin. Then, gently drying the crevicular site with an air syringe was carried out. Gingival crevice fluid (GCF) collection employed a single absorbent paper strip. The strip was gently inserted into the sulcus/pocket until slight resistance indicated proper placement. It remained undisturbed for 30 s to collect fluid. Any strips compromised by saliva or blood contamination were discarded. The MMP-9 was extracted from the absorbent paper by adding 100 ul of PBS, and then a vortex was done. After centrifugation for 10 min at 4000xg, the supernatant was used to quantify MMP-9 with an ELISA kit (ScienCell’s Human MMP9 ELISA Kit).

As for the periodontal assessment, the gingival index, plaque index, papillary bleeding scoring index and periodontal pocket depth were included in the present study:

1. Gingival Index (GI) described by Löe and Silness 1963 [27].
2. Plaque Index (PI) described by Silness and Löe 1964 [28].
3. The periodontal probe was inserted carefully into the gingival sulcus at the base of the interdental papillae mesial aspect. The intensity of bleeding is measured by rating on a scale of 0–4 [29]. The score criteria are 0: no bleeding, 1: one bleeding point appears, 2: multiple bleeding points or one bleeding line appears, 3: interdental triangle filled with blood, 4: excessive bleeding when probing and blood flow to the marginal sulcus. The bleeding amount will be calculated using the formula to find the PBI value. PBI is determined by dividing the amount of bleeding by the number of examined papillae.
4. Probing pocket depth (PPD), according to Ramfjord, 1967 [30].

Six measurements of PPD and CAL were taken around each tooth at specific locations: mesiobuccal, midbuccal, distobuccal, mesiolingual, midlingual, and distolingual. A UNC manual probe was used for these measurements. The values from all six sites were added and then divided by six to obtain an average score for each tooth in millimetres [30].

Both jaws were scanned using the intraoral scanner, which has an output of 7.7 Mw, a wavelength range of 400 to 700 nm, and a beam divergence of 10° (Planmeca Emerald S, Planmeca Oy, 00880 Helsinki, FINLAND).

**Fig. 2:**
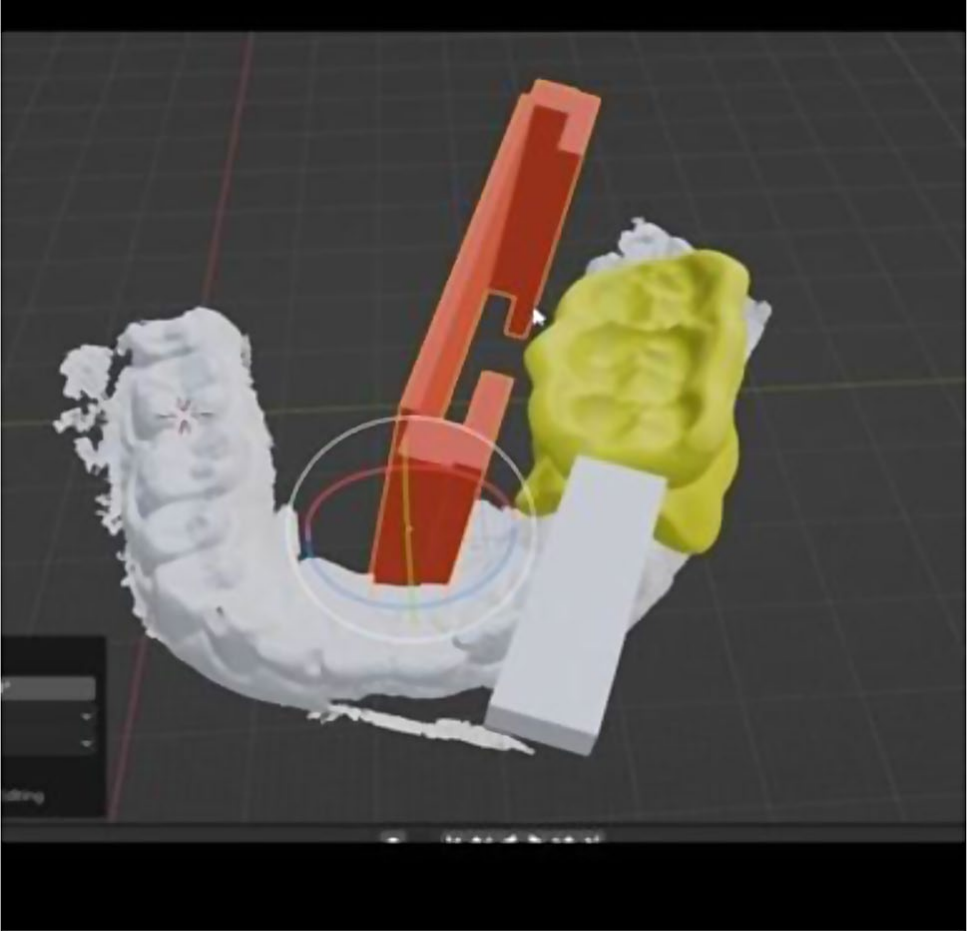
Steps of digital design of customized film holder

The meticulous design of the radiographic template was a two-step process. The first step involved designing a customized bite block for the patient, followed by designing a film holder. The custom bite block was designed using stent designing software (Real Guide 5.0 software, 3DIEMME) Fig. 2. v stent was then given a thickness of 2 mm and an occlusal thickness of 3 mm to prevent breakage from patients’ biting force. Finally, the stent was exported as a standard tessellation language file (STL).

The custom film holder was designed using a free modelling and designing program (Blender software V 2.83). It consists of a rectangular 4 cm length * 3 cm width to accommodate the film size, and two mesial and distal holding arms for the film are added.

**Fig. 3:**
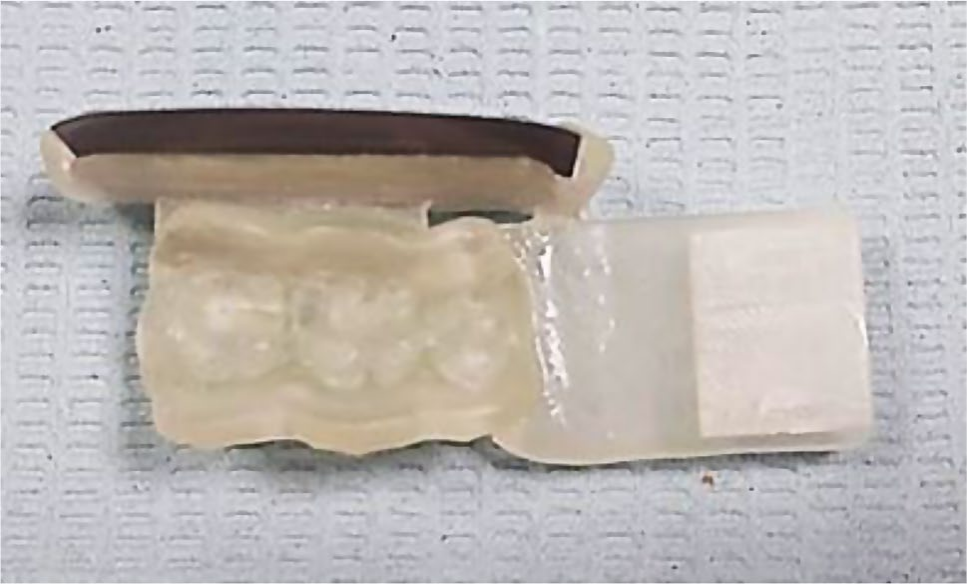
Customized radiographic template and film holder

The different parts of the assembly were attached using the Join software tool. Then, the designed assembly was 3D printed using clear stent resin (EPAX resin) using a Phrenzen Sonic mini 4 K 3D printer. After removing the supporting arms, the 3D-printed assembly was finished using a finishing stone, as shown in Fig. 3.

Digital bitewing radiographs were taken using a size 2 imaging plate (Guilin Woodpecker Medical Instrument Co, Ltd) and a dental x-ray machine ( Planmeca Prox, Planmeca Oy, 00880 Helsinki, FINLAND ) set at 60 kv, 8 mA, and 0.125s. The intra-oral radiographs were captured using the parallel long cone technique with a 16-inch cone. Two assessors assisted with the reliability of digital radiographs. Using Image J software (version 1.54) 580 × 454 pixels, RGB, 1 MB.

Evaluation parameters were:

1. Area of radiolucency at gingival seat interpreted as secondary caries.
2. Coronal periodontal ligament width.
3. Interdental crestal bone level.

Basic oral hygiene measures were instructed to the patients, brushing with fluoridated toothpaste twice and flossing daily. Eating a balanced diet and limiting snacks between meals. Assessments were done clinically, radiographically and biochemically at baseline (1 week) and after 3, 6 and 12 months. Data were collected, tabulated and statistically analyzed.

### Statistical analysis

Ordinal data were presented as frequency and percentage values. Numerical data were presented as mean and standard deviation values and were analyzed for normality using the Shapiro-Wilk’s test. Normally distributed data (MMP-9) were analyzed using an independent 0test for intergroup comparisons and repeated measures ANOVA followed by the Bonferroni post-hoc test for intragroup comparisons. Non-parametric numerical and ordinal data were analyzed using the Mann-Whitney U test for intergroup comparisons and Friedman’s test, followed by the Nemenyi post-hoc test for intragroup comparisons. Correlations were analyzed using Spearman’s rank-order correlation coefficient. Statistical analysis was performed with R statistical analysis software version 4.3.2 for Windows (R Core Team, 2023). R: A language and environment for statistical computing. R Foundation for Statistical Computing, Vienna, Austria. URL: https://www.R-project.org/.

## Results

**Fig. 4:**
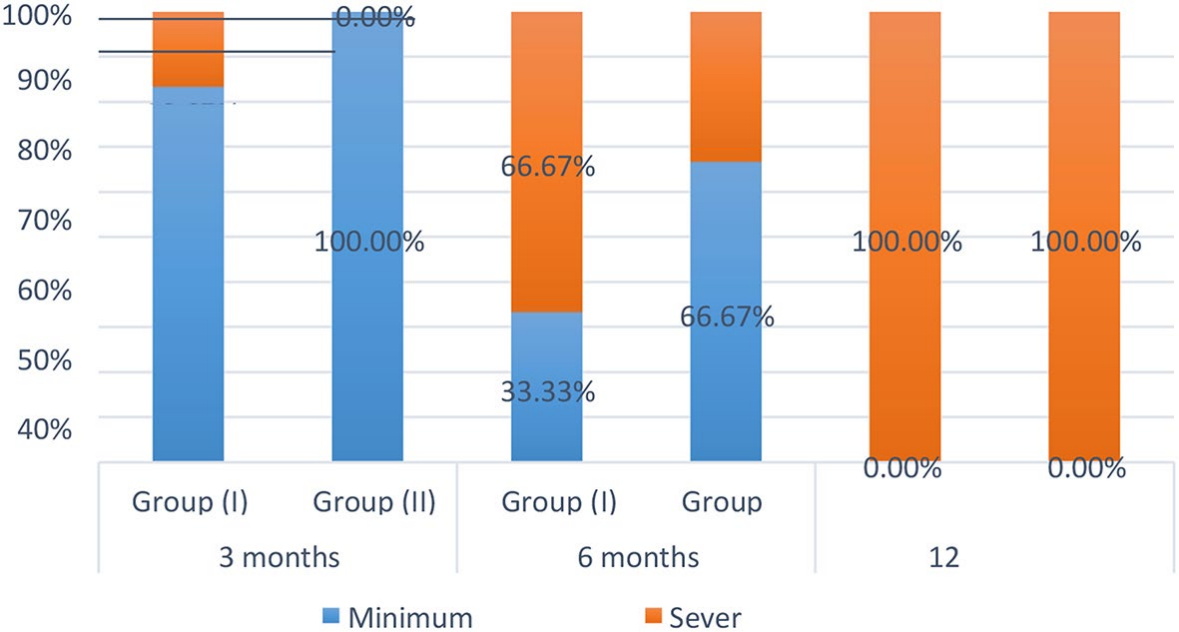
Stacked bar chart showing restorative evaluation

Results of inter- and intragroup comparisons for restorative evaluation are presented in Table 1 and Fig. 4. Results showed that at all intervals, there was no significant difference between the two groups (*p* > 0.05). For both groups, there was an important difference between values measured at different intervals, with the percentage of cases with severe extension found after 12 months being significantly higher than those measured after 3 and 6 months (*p* < 0.05).

**Fig. 5:**
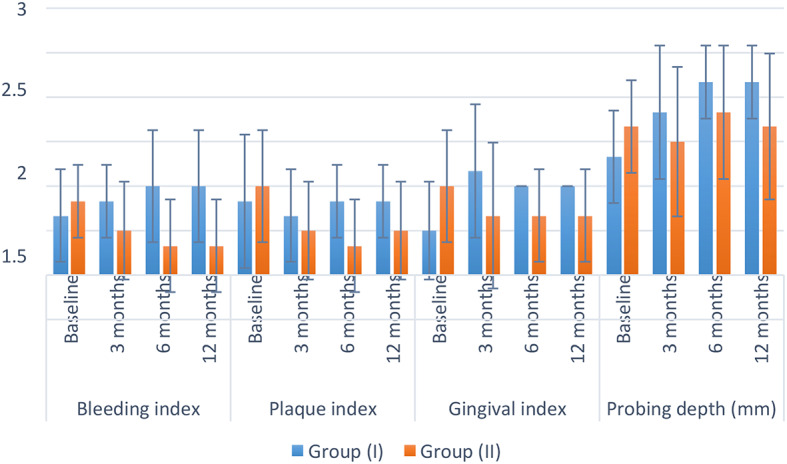
Bar chart showing mean and standard deviation (error bars) values for different periodontal parameters

Results of inter and intragroup comparisons for periodontal parameters are presented in Table 2 and Fig. 5. Results showed that within all measurements and intervals, there was no significant difference between both groups (*p* > 0.05). For group (I), there was a significant increase in measured probing depth after 6 and 12 months in comparison to baseline value (*p* < 0.001). While for all other measurements, the difference was not statistically significant (*p* < 0.05).

**Fig. 6:**
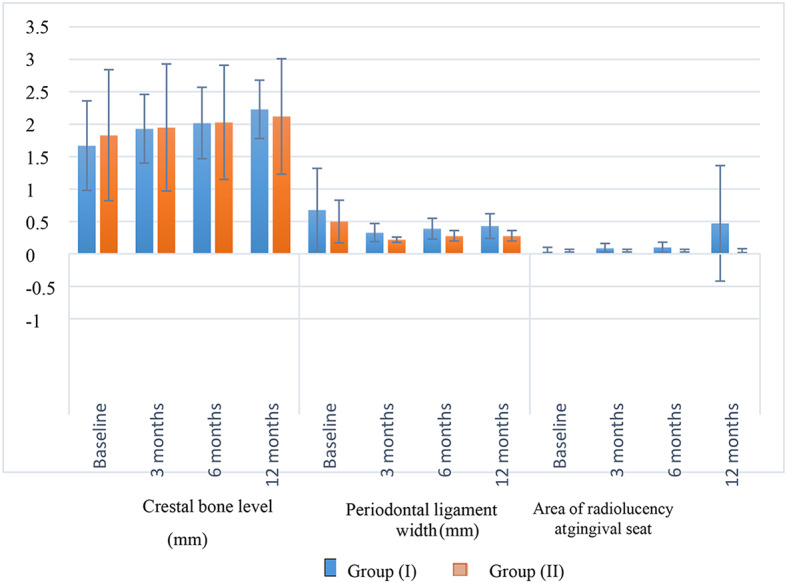
Bar chart showing mean and standard deviation (error bars) values for different radiological parameters

Table 3 and Fig. 6 present the inter- and intragroup comparison results for radiographic parameters. Results showed that for periodontal ligament width measured after 3 months, group (I) had a significantly higher value than group (II) (*p* = 0.018). For all other measurements, there was no significant difference between groups and between values measured at different intervals (*p* > 0.05).

**Fig. 7:**
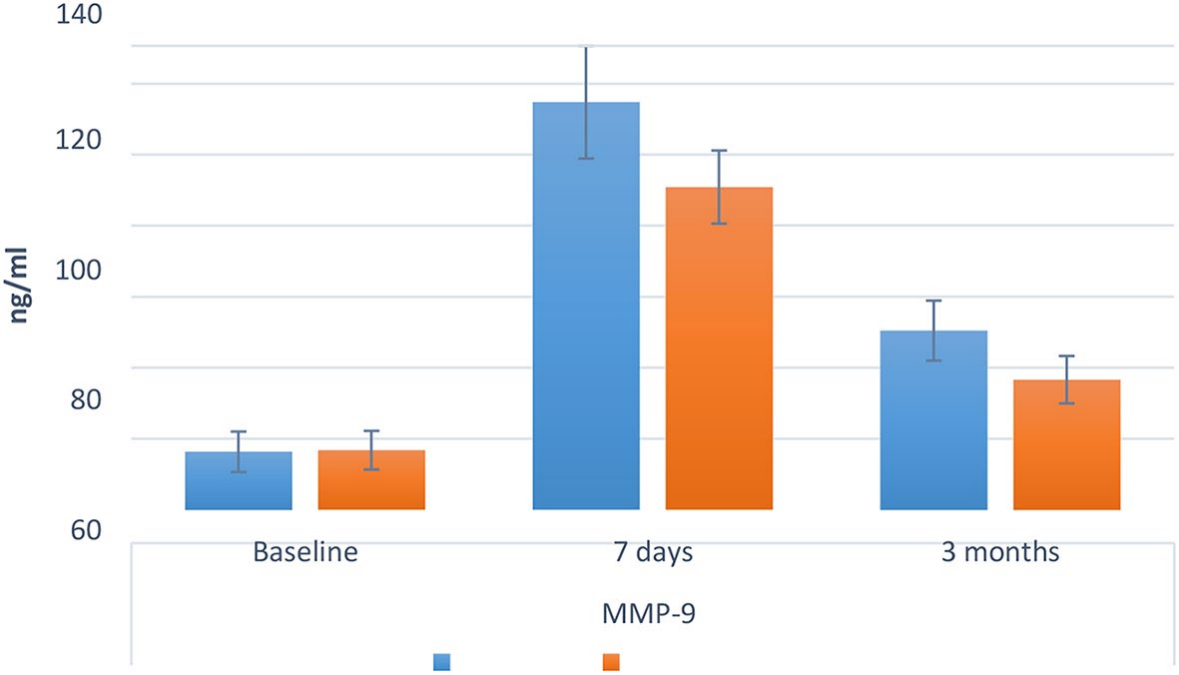
Bar chart showing mean and standard deviation (error bars) values for MMP-9

The inter and intragroup comparison results for MMP-9, presented in Table 4 and Fig. 7, have significant implications. Specifically, after 7 days and 3 months, group (I) had significantly higher values than group (II) (*p* < 0.05), while at baseline, the difference was not statistically significant (*p* = 0.897). Within both groups, there was an essential difference between values measured at different intervals, with the value measured after 7 days being the highest, followed by 3 months, and the value measured at baseline being the lowest (*p* < 0.001).

**Table 1:** Inter and intragroup comparisons of restorative evaluation

Time	Caries extension	*n* (%)		χ2	*p*-value
		Group (I)	Group (II)		
3 months	Minimum extension	50 (83.33%)^A^	60 (100.00%)^A^	**1.09**	**0.296**
	Sever extension	10 (16.67%)	0 (0.00%)		
6 months	Minimum extension	20 (33.33%)^A^	40 (66.67%)^A^	**1.33**	**0.248**
	Sever extension	40 (66.67%)	20 (33.33%)		
12 months	Minimum extension	0 (0.00%)^B^	0 (0.00%)^B^	**NA**	**NA**
	Sever extension	60 (100.00%)	60 (100.00%)		
χ2		**7.60**	**9.33**		
*p*-value		0.022*	0.009*		

Values with different superscript letters within the same vertical column are significantly different

*Significant (*p* < 0.05)

**Table 2:** Inter and intragroup comparisons of different periodontal parameters

Parameter	Interval	Mean ± SD		Test statistic	*p*-value
		Group (I)	Group (II)		
Bleeding index	Baseline	0.67 ± 0.52 ^A^	0.83 ± 0.41 ^A^	**21.00**	**0.595**
	3 months	0.83 ± 0.41 ^A^	0.50 ± 0.55 ^A^	**24.00**	**0.282**
	6 months	1.00 ± 0.63 ^A^	0.33 ± 0.52 ^A^	**28.00**	**0.091**
	12 months	1.00 ± 0.63 ^A^	0.33 ± 0.52^A^	**28.00**	**0.091**
	Test statistic	**2.54**	**7.20**		
	p-value	**0.468**	**0.066**		
Plaque index	Baseline	0.83 ± 0.75 ^A^	1.00 ± 0.63^A^	**20.50**	**0.718**
	3 months	0.67 ± 0.52 ^A^	0.50 ± 0.55 ^A^	**21.00**	**0.640**
	6 months	0.83 ± 0.41 ^A^	0.33 ± 0.52 ^A^	**27.00**	**0.112**
	12 months	0.83 ± 0.41 ^A^	0.50 ± 0.55 ^A^	**24.00**	**0.282**
	Test statistic	**0.29**	**5.55**		
	p-value	**0.962**	**0.136**		
Gingival index	Baseline	0.50 ± 0.55 ^A^	1.00 ± 0.63 ^A^	**25.50**	**0.201**
	3 months	1.17 ± 0.75 ^A^	0.67 ± 0.82 ^A^	**24.50**	**0.306**
	6 months	1.00 ± 0.00 ^A^	0.67 ± 0.52 ^A^	**24.00**	**0.174**
	12 months	1.00 ± 0.00 ^A^	0.67 ± 0.52 ^A^	**24.00**	**0.174**
	Test statistic	**5.18**	**3.00**		
	p-value	**0.159**	**0.392**		
Probing depth (mm)	Baseline	1.33 ± 0.52^B^	1.67 ± 0.52 ^A^	**1.12**	**0.290**
	3 months	1.83 ± 0.75^AB^	1.50 ± 0.84 ^A^	**0.73**	**0.485**
	6 months	2.17 ± 0.41 ^A^	1.83 ± 0.75 ^A^	**0.95**	**0.363**
	12 months	2.17 ± 0.41 ^A^	1.67 ± 0.82 ^A^	**1.34**	**0.209**
	Test statistic	**9.57**	**0.36**		
	p-value	< 0.001*	**0.785**		

Values with different superscript letters within the same vertical column are significantly different

*Significant (*p* < 0.05)

**Table 3:** Inter and intragroup comparisons of different radiographic parameters

*Parameter*	*Interval*	*Mean ± SD*		*Test statistic*	*p*-value
		*Group (I)*	*Group (II)*		
Crestal bone level (mm)	Baseline	1.67 ± 0.69	1.83 ± 1.01	**17.50**	**1**
	3 months	1.93 ± 0.53	1.95 ± 0.98	**16.50**	**0.872**
	6 months	2.02 ± 0.55	2.03 ± 0.88	**16.00**	**0.809**
	12 months	2.23 ± 0.45	2.12 ± 0.89	**22.00**	**0.570**
	Test statistic	**1.19**	**0.18**		
	p-value	**0.348**	**0.908**		
Periodontal ligament width (mm)	Baseline	0.68 ± 0.64	0.50 ± 0.33	**18.50**	**1**
	3 months	0.33 ± 0.14	0.22 ± 0.04	**32.50**	0.018*
	6 months	0.39 ± 0.16	0.28 ± 0.08	**25.50**	**0.256**
	12 months	0.43 ± 0.19	0.28 ± 0.08	**29.50**	**0.062**
	Test statistic	**1.18**	**3.03**		
	p-value	**0.350**	**0.062**		
Area of radiolucency at gingival seat	Baseline	0.06 ± 0.04	0.05 ± 0.02	**24.00**	**0.375**
	3 months	0.09 ± 0.07	0.05 ± 0.02	**24.00**	**0.375**
	6 months	0.10 ± 0.08	0.05 ± 0.02	**24.00**	**0.375**
	12 months	0.47 ± 0.89	0.05 ± 0.03	**28.00**	**0.126**
	Test statistic	**1.24**	**0.36**		
	p-value	**0.331**	**0.781**		

*Significant (*p* < 0.05)

**Table 4:** Inter and intragroup comparisons of MMP-9

Interval	Mean ± SD (ng\ml)		Test statistic	*p*-value
	Group (I)	Group (II)		
Baseline	16.39 ± 5.70^C^	16.82 ± 5.43^C^	**0.13**	**0.897**
7 days	114.75 ± 15.87^A^	90.89 ± 10.28^A^	**3.09**	0.011*
3 months	50.50 ± 8.43^B^	36.67 ± 6.68^B^	**3.15**	0.010*
*Test statistic*	**149.47**	**180.37**		
*p-value*	< 0.001*	< 0.001*		

Values with different superscript letters within the same vertical column are significantly different, *Significant (*p* < 0.05)

## Discussion

One of the significant challenges facing resin composite materials is polymerization shrinkage, which causes gap formation at the tooth restoration interface, followed by recurrent caries. For optimal bonding between composite material and tooth structure, dentists frequently employ a layering technique to apply the composite in layers to eliminate gap formation [31].

The protocol of incremental filling is accepted as a gold standard method to assure adequate curing depth and minimize polymerization shrinkage stresses. However, this procedure is time-expendable as successive layers are to be laid and cured. Also, air void entrapment between layers and technique sensitivity are considered apparent problems [32].

The simplification of daily clinical procedures is necessary, so the bulk-fill technique was released to help clinicians who prefer to adopt simple techniques that allow the insertion of cavity filling in larger increments and shorter chair time [33]. 

To allow placing one significant increment of 4–5 mm thickness, the molecular structure of bulk-fill composites was customized by incorporation of stress relievers and high molecular weight monomers, as monomers with low molecular weight allow a higher degree of conversion due to promoting a higher number of double bonds per unit of weight [34]. The main advantage of bulk-fill composites is increased curing depth due to high translucency, more effective initiator systems, and low polymerization shrinkage due to modifications in the organic matrix or filler content [35].

Xtra Fill resin composite was the material of choice in the current study; the superiority of Xtra Fill was related to their resin matrix composition, which contains low viscosity monomers TEGDMA, which increases the flow and decreases the viscosity of the resin matrix, therefore increasing the degree of conversion with low shrinkage stresses which affect the tooth – restoration interface leading to recurrence of caries [36]. It has superior translucency despite its high filler loading due to the increased filler size; the refractive index of the resin matrix and the filler particles are improved [37].

Restorations were inserted employing two filling techniques in cavities prepared in the teeth of different quadrants of each patient; both groups were exposed to comparable environmental, mechanical, and oral hygiene circumstances. Since the variables impacting the clinical result depend more on the operator’s technique than on the material tested, only one experienced operator (M.M.T) inserted all the restorations. This guaranteed that the restorations were operated at the same settings. This reduced the probability of bias. Accordingly, the results would be affected only by the different restorative techniques.

Restorations were examined visually, probing and flossing for any roughness or irregularities.

Assessing six sites in every tooth [38]. This method enables accurate assessment of the frequency and severity of periodontal disease. When paired with multilevel analysis, it allows consideration of the results at particular sites while accounting for the correlation between sites from the same tooth and teeth belonging to the same patient.

Furthermore, bitewing radiography is a valuable method for determining the existence of proximal caries and periodontal health. Technical errors during radiography result in inaccurate radiographic interpretation, poor diagnosis, and unnecessary patient exposure to radiation due to the great possibility of retakes.

The expanding application of digital technology in dentistry is improving the recording of occlusal contact. Digital intraoral scans present superior time efficiency [39]. The customized radiographic template and film holder designed and fabricated in the current study make bitewing radiography more standardized and reproducible.

Considering the decreased rate of errors utilizing the newly designed custom-made holder, it can be recommended to be used in daily dental clinical practice, resulting in fewer radiographs and, eventually, a lower patient radiation dose. As previously proven, compared to the ordinary film holder, the ready-made modified one reduced the horizontal overlapping of the teeth and wrongly positioned films [40].

Regarding the results of the current study, although the incremental packing technique gave a better result in terms of recurrent caries, no significant differences appeared between incremental and bulk-filled posterior restorations, so the null hypothesis was accepted clinically.

This agrees with a previous study [41], which found that both techniques showed comparable clinical efficacy on posterior resin composite restorations. Current results agreed with another study [33], which stated that, through various assessed follow-up periods, posterior restorations done using the bulk-filling technique exhibit satisfactory clinical performance comparable to restorations done using the typical incremental technique. Some studies [24, 32, 42] agreed with our results and concluded that no statistically significant differences in marginal gap formation were observed when comparing bulk-fill with incremental application techniques, supporting both techniques’ similar behaviour and survival rate after 12, 36-month follow-up.

The results of the present study disagreed with a study [33] that found that incrementally placed resin composite material had better internal adaptation to cavity walls than bulk-placed material.

Another study [43] compared the adaptability of composites applied by both techniques and found that bulk-fill resin composites showed better adaptation and less marginal leakage than conventional incremental restorations.

In both groups, a significant difference was recorded between results collected at different follow-up periods, with the percentage of cases with severe extension found after 12 months being higher significantly than results measured after 3 and 6 months. Excessive polymerization contraction stresses at the tooth-restoration interface may lead to marginal defects. Deterioration at the resin/bond interface due to slow hydrolysis may contribute to the rise in marginal flaws over investigation times to statistically significant levels [44]. More than 700 different species of natural oral habitats of microorganisms, with their complicated biochemical activities, enzymatic and acidic products ordinarily exist. These influencing factors interact together to degrade dental restorations [22] gradually. The contact strength of proximal restorations is not stable; this may be due to proximal wear, mesial movement or the change over time caused by material biodegradation [45]. This may lead to increased opportunities for food impaction and complicated interproximal hygiene measures.

**Fig. 8:**
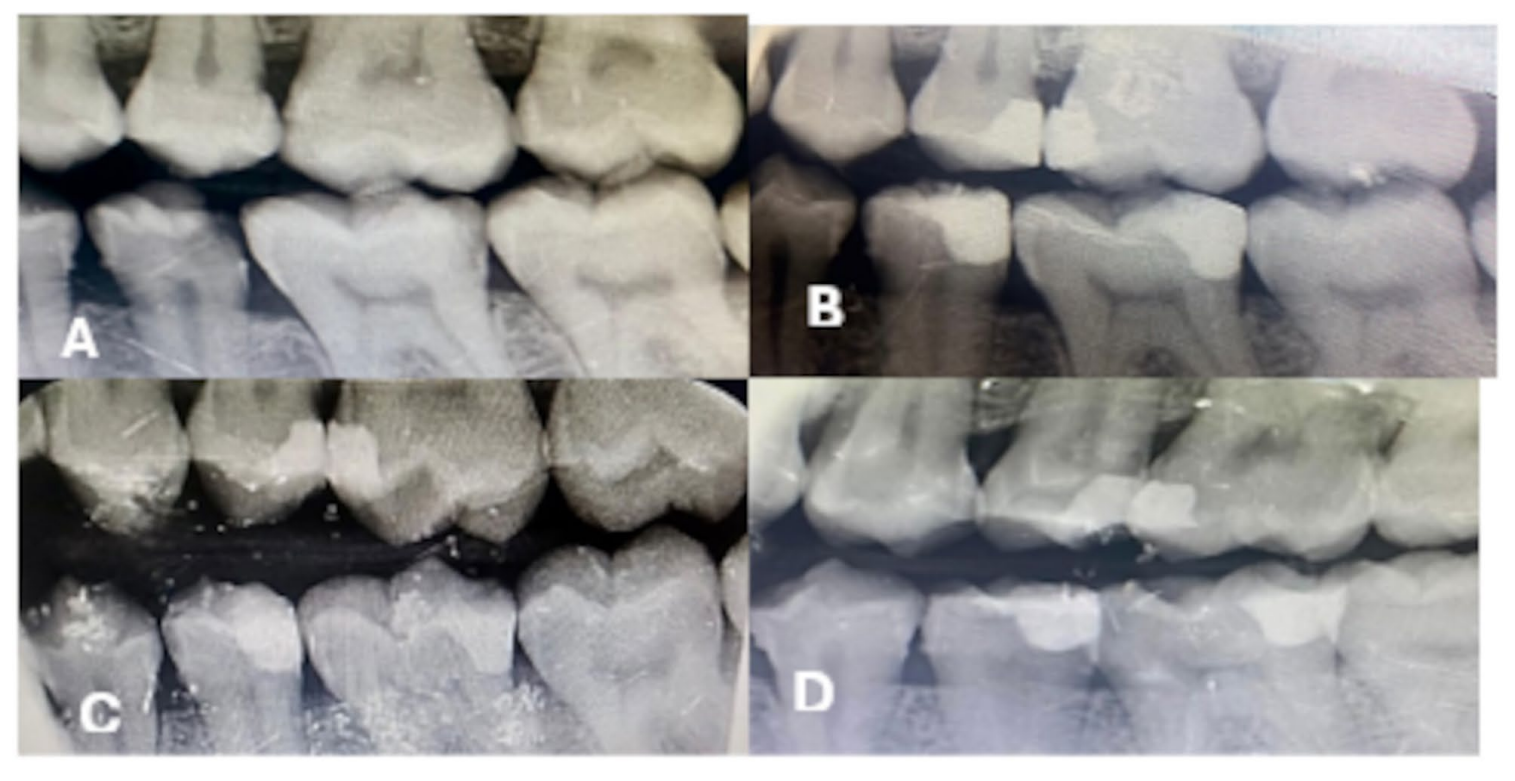
(**a**): Base line, (**b**): follow up after 3 months, (**c**): after 6 months, (**d**): after 12 months

The null hypothesis in radiographic assessment was accepted except for Periodontal ligament width at 3 months; the non-significant results between both radiographic techniques can be explained by the small sample size, as radiolucency, Fig. 8, the radiographic appearance of demineralization, can be influenced by many factors like exposure parameters, type of image receptors, display systems and viewing condition. Optical illusions like cervical radiolucency (burn-out effect) might be falsely interpreted as carious lesions [46, 47].

The null hypothesis for periodontal assessment was accepted. A previous study [48] proved that restorations might harm periodontal health. A significant association between the presence of proximal restorations and the prevalence of periodontal disease was observed.

Following our results, another study [49] highlighted negligible, non-significant statistical differences in the degree of inflammation after restorations, possibly due to patients, tooth and case selection, and supportive measures. They also concluded that composite resin exhibits good biocompatibility, as no significant differences were found in the inflammation between control and treatment tissues.

Another study [50] disagreed with our findings, claiming that, up to a year of follow-up, the clinical and molecular risk of periodontal tissue healing was associated with Iuxta/subgingival interproximal restorative margins.

A cohort clinical study [38] partially confirms the assumption that periodontal disease is correlated with proximal restorations. Although not indicating a disease, a significant increase was detected in clinical attachment loss (CAL) and probing depth (PD) at restored sites.

Matrix metalloproteinases (MMPs) are a group of Ca and Zn ions dependent enzymes which have the power to degrade extracellular components and play a significant role in some biological and pathological processes. They comprise a family of around 25 members broadly categorized into six groups of MMPs recognized in humans, which include gelatinases, collagenases, matrilysins, stromelysins, and membrane-type [51]. MMPs are crucial to the formation of teeth. About 65% of dentine comprises inorganic minerals, and the remaining 35% is organic matrix, comprising 10% noncollagenous proteins and 90% type I, III, and V collagens in detail [22].

Once the dentin matrix is mineralized, these enzymes are trapped within the apatite crystals, becoming structurally stable and non-functional. As long as dentin is mineralized, these enzymes stay inactive. However, due to caries by microbial acids or during acidic etching before bonding, intense enzymatic activity and autodegradation of the hybrid layer can occur upon demineralization of dentin [51, 52].

During the progression of the caries process, dentin demineralization is followed by a breakdown of the collagenous organic matrix of dentin, evidently not by bacterial action; it has been recently thought to be mediated mainly by host-derived MMPs. Also, the acidic environment created by bacterial acids can facilitate the activation of endogenous MMPs [52].

Another study claimed that Bacterial collagenases plus endogenous MMPs of saliva, gingival fluid and dentinal origin contribute to dentine matrix degradation in active carious lesions [22]. Moreover, there is a significant relationship between levels of MMP-9 in shallow and deep caries. It is reported to be dominant in dentin caries lesions, especially in the outer affected caries layer if compared to the inner caries layer [52], which explains that in proximal carious lesions, MMPs derived from both gingival crevicular fluid and salivary glands can effectively degrade exposed dentinal collagen matrix. Another study supports that salivary MMPs significantly involve dentine matrix degradation during the carious process [53].

During the bonding procedure, the acidic nature induced by adhesives also provides excellent circumstances for initiating MMPs; acidic activation induced by etch-and-rinse and self-etch adhesives has an additional influence on the rise of MMP-2 and − 9 protein levels [52].

Another contributing factor is the deficiency of TIMPs’ inhibitory action. TIMPs are endogenous inhibitors attached to MMPs, and the balance between them is essential for maintaining the inhibitory activity of healthy tissues. Mildly acidic resin monomers can activate MMPs by stopping the action of tissue inhibitors of metalloproteinases-1 (TIMP-1) [52].

Recurrent caries at the gingival seat start in areas of released gingival fluid. MMPs exist in gingival crevicular fluid and radicular dentine, permitting these lesions. Another author [54] also stated that within the limitations of their study, MMP-9 had some changes in crevicular fluid over time, with no evident relation to the bonding technique or type of restorative materials.

MMP-9 is synthesised mainly by macrophages and neutrophils and is used in MMP-mediated destructive periodontal disease. Epithelial cells release high levels of matrix metalloproteinases (MMPs). These MMPs cause the junctional epithelium to move upwards and outwards, ultimately leading to connective tissue detachment [55]. This highlights the well-known importance of MMPs in gum disease (periodontal disease) [22].

Several studies [17, 56] have demonstrated that the presence of the bacterium Porphyromonas gingivalis is a culprit in gum disease by promoting the movement of immune cells called monocytes. This happens because the bacteria trigger the production of an enzyme, MMP-9, which breaks down tissue barriers. This increased MMP-9 activity, often found in people with gum disease, allows monocytes to migrate and participate in inflammatory responses, potentially leading to tissue destruction.

Another study stated that (MMP-9), is related to the degradation of collagen and gelatin in the gingival crevicular fluid and is distinctly elevated in periodontitis [57].

These findings may explain the current results to a limited extent. The literature has limited studies assessing the existence of MMPs in gingival crevicular fluid in teeth restored with proximal composite restorations, so comparing our results with those of previous studies is difficult. The null hypothesis for biochemical assessment was rejected as there were significant changes in levels of MMP-9 at different testing times in the current study.

Some articles have exhibited upregulated MMP-9 in gingival crevicular fluid (GCF) during the early stages of periodontitis, which may consider MMP-9 a predictor of early disease activity.

One of the study’s limitations is the range of ages from 18 to 50, a crucial period when people may start experiencing age-related severe dental problems. Many factors, such as systemic diseases, oral hygiene, hormones and habits, may influence their dental condition. Secondly, it is worth mentioning that clinical, visual and tactile examination of gingival margins of posterior proximal restorations for detecting recurrent caries is a complex procedure that may only be partially useful, and good radiographic images for accurate follow-up must support findings.

This is the first clinical study correlating restorative treatment with periodontal, radiographic, and MMP-9 levels. Further clinical studies with different subjects and study designs are recommended to investigate the correlation between different follow-up methods for recurrent caries in proximal composite restorations.

## Conclusions

According to the conditions of the current study, it could be concluded that:

1. The bulk fill technique can be considered an efficient alternative to the incremental fill technique in restoring proximal cavities. It produces similar results but is less sensitive and time-saving.
2. Early evidence of caries recurrence can be correlated to an increase in MMP-9 level in gingival crevicular fluid followed by an increase in radiographic periodontal ligament width measurement.

### Clinical relevance

1. MMP-9 level could be recommended as a chairside test for early prediction of caries recurrence in proximal composite restorations.
2. MMP-9 level is a practical biomarker for detecting periodontitis and a helpful tool for follow-up.
3. Anti-collagenolytic agents are recommended to be used to counteract the destructive effects of MMPs.
4. The custom-made radiographic template is an easy and valuable method of standardising and reproducing radiographic techniques.

## Data Availability

The datasets used and/or analyzed during the current study are available from the corresponding author upon reasonable request.
